# The poet Ulrich von Hutten (1488–1523) and the French disease: the records and human remains of a probable yaws patient

**DOI:** 10.1017/mdh.2024.38

**Published:** 2025-01

**Authors:** Urs Leo Gantenbein

**Affiliations:** Institute of Evolutionary Medicine, University of Zurich, Zurich, Switzerland

**Keywords:** Ulrich von Hutten, Yaws, Syphilis, Paleopathology, Renaissance, Humanism

## Abstract

Ulrich von Hutten (1488–1523), a renowned sixteenth-century German humanist, documented the symptoms of the epidemic that swept through Europe starting around 1495, commonly known as the French Disease. While it has traditionally been associated with venereal syphilis, Dutch tropical physician Willem F. R. Essed proposed in 1933, largely unnoticed to this day, that this new disease might instead be tropical yaws. This study establishes a clear link between Hutten’s reported symptoms and yaws, especially in its secondary and tertiary stages. The skeleton discovered in 1968 on Ufnau Island in Lake Zurich where Hutten died and was buried, exhibits distinct bone manifestations of ancient treponematosis with a pattern more consistent with yaws than syphilis. Furthermore, the correspondence between Hutten’s main symptoms and the lesions observable on the 1968 skeleton further confirms the identification of these human remains. The historical evidence of yaws significantly contributes to our understanding of this early modern epidemic.

## A life of quest and strife

Two entirely distinct worlds clashed within the person of the German humanist Ulrich von Hutten (21 April 1488–29 August 1523). On one hand, he was the proud descendant of an influential Franconian family of minor nobility, upholding the ancient ideals and privileges of imperial knighthood and asserting their rights through force during personal feuds. On the other hand, he was a gifted poet and learned humanist who sought to reform the traditional political and religious systems. Hutten fell victim to one of the most devastating epidemics that swept across Europe during the Renaissance. Initially most commonly known as the French Disease, it emerged on a significant scale in the French army during their occupation of Naples in the summer of 1495.^1^ For a considerable period, it was commonly believed to be sexually transmitted syphilis, with Hutten’s perceived moral degeneracy often highlighted. However, relying on Hutten’s detailed account of his illness and the observed changes in his human remains, there is substantial evidence indicating that he did not suffer from venereal syphilis but rather from closely related yaws.

Within the scope of this study, we will offer only a concise overview of Hutten’s life.^2^ As a humanist, he was drawn into an intellectual movement that originated in Italy and gained momentum in Germany in the mid-fifteenth century.^3^ It embraced the rediscovery of ancient literature and found its expression mainly in rhetoric, poetry, history, and moral philosophy. Germany did not become a single nation until 1871. It was a collection of small states ruled by local leaders, loosely united as the German Empire. The Roman Curia’s control, involving fees and heavy taxes, caused dissatisfaction long before Luther. Hutten’s aim in life was to challenge the Roman Curia’s authority and unite Germany under a strong emperor, but it ended tragically in failure. Against the wish of his father, Hutten’s early passion for intellectual pursuits led him to enrol in universities such as Mainz, Cologne, Erfurt, and Frankfurt on the Oder, where he earned his bachelor’s degree in 1506. Eager to deepen his humanistic skills, Hutten ventured to the University of Greifswald in the autumn of 1509. There he soon clashed with law professor Henning Lötze and his influential family. Initially, the Lötzes had supported Hutten, but in the heart of winter, just as he was preparing to leave for Rostock, they orchestrated a robbery that left him destitute. This pivotal incident, narrated from Hutten’s perspective, inspired him to compose his first significant Latin poem, his *Querelae* (complaints) against the Lötzes (1510).^4^ This poem not only showcased Hutten’s exceptional humanistic and poetic talents but also unveiled the simmering anger of a knight wronged, who sought vengeance through his literary feud. The poem also includes significant details about the gradual onset of his treponemal disease, as we will explore further below.

In 1511, his journey took him to Vienna, where he encountered a vibrant circle of patriotic humanists. The proximity to the imperial court sparked a newfound interest in politics, which would soon play a central role in his life. Following his first visit to Italy from 1512 to 1514, Hutten secured a position as a secretary at the court of the Archbishop of Mainz. After his second sojourn in Italy from 1515 to 1517, Hutten achieved his most significant personal triumph by earning the prestigious title of poet laureate from Emperor Maximilian, which enabled him to teach rhetoric and poetics. The growing discontent within Germany regarding Roman authority gained significant momentum with the emergence of Martin Luther in 1517. A critical moment arrived with the death of Emperor Maximilian in 1519, who had supported the German cause. His successor, the young Emperor Charles V, leaned more towards the position of the Roman church. Initially, Hutten held hopes for the new emperor. He envisioned the dismantling of Rome’s authority and that of the local princes, with the emperor and the knights playing a central role. Against this backdrop, Hutten created a series of Latin dialogues in which he vigorously opposed the Roman Curia. Influenced by Luther’s German-language writings, Hutten also transitioned to writing in German in 1520. He saw himself as an essential part of the rightful struggle alongside Luther against the ‘tyranny’ of the Pope and the Curia.

However, all of these literary attacks on Rome did not go unanswered. Since the end of 1520, a papal decree demanded the arrest and condemnation of Hutten as a heretic. In a military campaign against Duke Ulrich of Württemberg, Hutten met the powerful army commander Franz von Sickingen (1481–1523), whom Hutten was able to win over to his political tenets. Hutten sought refuge at Sickingen’s castle Ebernburg in September 1521, from where he embarked on an intense propaganda campaign for his cause. However, Charles V remained steadfast in his support of Rome. On 8 May 1521, at the Diet of Worms, he issued the imperial ban against Luther. Hutten’s dream of a reform of Germany under the emperor’s leadership seemed shattered. For a brief period, Hutten attempted to advance his plans independently, issuing a call for a ‘Pfaffenkrieg’ (Clergy War) in a manner reminiscent of petty-minded robber knights.^5^ Sickingen’s attempt in September 1522 to achieve the desired comprehensive reform by forcibly seizing the Electorate of Trier ended in a dismal failure and cost him his life.

Hutten found himself with no alternative but to seek refuge in Switzerland in November 1522. He ultimately passed away on the little Ufnau (or Ufenau) Island in Lake Zurich by the end of August 1523. After his death, Hutten only gradually became an integral symbol of German national identity.^6^ It was not until the late eighteenth century that Hutten was perceived as the catalyst for the awakening of the German nation and as a martyr for freedom. Continuously reshaped and reinterpreted, Hutten became a figure onto which each era and ideology projected its own ideals. As a result, the prevailing image of Hutten became more ideologically influenced than historically accurate.

The current standard edition of Hutten’s complete works is the one published by Eduard Böcking (1802–70) in seven volumes between 1859 and 1870.^7^ However, this edition falls short of modern standards and poses challenges due to numerous shortcomings.^8^ Aside from all comments being in Latin, it has an idiosyncratic and inconsistent structure, mixing peripheral or even superfluous texts with essential ones. Furthermore, it is problematic that in Böcking’s edition, the preface and main part of the text appear separated in different volumes, disrupting the integrity of the respective works. Another shortcoming in the realm of modern Hutten studies is that a comprehensive scholarly biography of Hutten is still missing. The philosopher and theologian David Friedrich Strauss (1808–74) stands out with his extensive biography, first published in 1858 and a second revised edition in 1871 with multiple reprints, which was characterised by a detailed, factual richness.^9^ However, this was not devoid of contemporaneous ideological notions. Modern Hutten studies will aim to finally establish a sober and scholarly foundation for his biography and the new edition of his works.

## Towards understanding the early epidemic of the French Disease

Despite the detailed source studies by medical historians Arrizabalaga, Henderson, and French (1997), which emphasised the divergence of early reports and cautioned against equating the French Disease with venereal syphilis, this probable misconception persists in scientific publications discussing the possible origins of treponematoses.^10^ In the same vein, McGough (2010) has emphasised that in historical studies of this early modern epidemic and its aftermath, the modern term ‘syphilis’, with all its associated connotations, should be avoided wherever possible.^11^ Contemporaries typically referred to the new disease as the ‘French Disease’, associating it not only with its presumed origin in the invading French army but also with moral concerns, unfavourable astrological constellations, or simply the notion of divine punishment. It should also be remembered that the symptom complex of the French Disease could encompass not only syphilis or, as we shall see, yaws, but also other sexually transmitted diseases such as gonorrhoea, or even entirely different skin conditions.

In 1905, Fritz Schaudinn (1871–1906) and Erich Hoffmann (1868–1959) discovered the pathogen responsible for venereal syphilis, naming it *Spirochaeta pallida.* ^12^ This organism is today recognised as a subspecies called *Treponema pallidum* ssp. *pallidum* (TPA). Shortly thereafter, in the same year, Aldo Castellani (1874–1971) identified a closely related pathogen causing non-venereal yaws, which he named *Spirochaeta pertenuis*, now classified as *Treponema pallidum* (T.p.) ssp. *pertenue* (TPE).^13^ Additionally, two other endemic treponematoses are not sexually transmitted, namely, bejel, caused by the pathogen *T. p. endemicum* (TEN) and the rare pinta, caused by *T. p. carateum.* However, we will not delve into these diseases here.^14^ Even in modern times, the clinical presentations of venereal syphilis and yaws, especially their skin manifestations, are so similar that they are often mistaken for one another.^15^ Based on a comprehensive review of early documentation of the French Disease, Dutch tropical physician Willem F. R. Essed (1893–1939) proposed, in his 1933 doctoral thesis titled *Over den Oorsprong der Syphilis* [On the Origin of Syphilis], the theory that the new plague haunting early modern Europe was caused more by yaws than by venereal syphilis.^16^ Despite being published as a book in Dutch, Essed’s significant findings have received limited attention in research up to the present day.^17^ Recent paleogenetic investigations seem to support this hypothesis, at least partly. In a study conducted by Giffin, Lankapalli, Sabin, et al. (2020), ancient DNA of yaws was discovered in the remains of a post-medieval plague victim of Vilnius, Lithuania. As a result, the authors infer that yaws could have been an important contributor to the sudden epidemic in late fifteenth-century Europe. They even observed that Hutten’s description of ‘welts the size of acorns’, which we will discuss further below, might correspond to the raspberry-like ulcers characteristic of the early stage of yaws.^18^ Moreover, in genetic data from early modern Northern European human remains, Majander et al. (2020) found a diversity of *T. pallidum* strains, including those associated with venereal syphilis and yaws. The yaws strain was discovered in Turku, Finland. In addition, a previously unknown *T. pallidum* lineage from the island of Sylt in northern Germany was also discovered as a sister group to yaws- and bejel-causing lineages. The authors conclude that the early treponemal epidemic pattern was more complex than previously understood.^19^

Several competing theories have been proposed to explain the evolutionary history of treponematoses, particularly the major early modern epidemic since around 1495. However, the validity of these hypotheses remains largely unclear and subject to debate.^20^ One of the three most significant theories is the Columbian hypothesis, which suggests that syphilis originated in America and was brought back by Columbus on his return from his first voyage in 1493. In contrast, the pre-Columbian hypothesis argues that syphilis existed in the Old World long before Columbus but was often confused with other skin diseases such as leprosy. The Unitarian hypothesis, first proposed by Ellis Herndon Hudson (1890–1992) in 1963, posits that all forms of treponematoses, whether venereal or non-venereal, are caused by the same pathogen, *Treponema pallidum*. According to this theory, the distinct clinical manifestations, such as venereal syphilis, yaws, endemic syphilis, and pinta, represent different ecotypes of a fundamentally uniform pathogen that is primarily influenced by climatic, social, and demographic factors where the disease is contracted.^21^ However, recent paleogenetic results indicate a significantly more intricate global distribution pattern of treponematoses than previously hypothesised, suggesting differentiation into various manifestations of *Treponema pallidum* dating back hundreds or thousands of years, along with a longstanding coexistence of venereal syphilis and yaws.^22^ While not all questions have been resolved, further new findings are anticipated.

The case of Ulrich von Hutten offers a unique opportunity as it provides a detailed description of an early sufferer of the French Disease, allowing for a comparison of his symptoms with the pathological skeletal changes evident in his remains. The primary objective of this study is to directly translate Hutten’s multifaceted account from Latin and interpret it accordingly. The principal finding suggests a departure from previous assumptions, proposing that Hutten likely did not suffer from venereal syphilis, but rather from yaws or a closely related variant that remains unidentified. Arrizabalaga (2002) rightly pointed out the challenges of retrospective diagnoses from historical case reports, particularly when historical sources use vague terms differing from modern usage.^23^ Furthermore, medical historians from the emerging era of laboratory medicine often yielded to the temptation of retroactively applying modern disease concepts to historical case descriptions. So, it is problematic to assume, particularly in light of the Unitarian hypothesis, that a tropical disease like yaws would have presented exactly the same way centuries earlier in a completely different geographical, climatic, social, and demographic environment. Additionally, it is unreasonable to suppose that the disease would not have undergone fundamental genetic changes over time. Nevertheless, Hutten’s case offers us a unique comparative opportunity.

As a layman, Hutten’s reports lack technical terminology, relying instead on generally understandable and precise terms such as pain, swellings, ulcers, discharges, and their localisation. This enables a comparison of his symptoms with known clinical presentations, facilitating the formulation of a tentative diagnosis or at least a differential diagnosis. If the modern symptoms closely match the historical ones, as observed in the case of Hutten, then it becomes pertinent to consider the possibility of a retrospective diagnosis. Moreover, it is crucial to consider that the manifestation and severity of an infectious disease are influenced not only by the pathogen’s virulence but also by the microbial load and the host’s acquired immunity.^24^ In this treponemal epidemic, these factors may have exerted an even more significant influence than changing environmental conditions. Although yaws today is predominantly a childhood disease due to early immunity acquisition, it could have severely impacted an unprepared population.

In Hutten’s case, a retrospective diagnosis is further supported by the paleopathological findings. As we will see below, according to Steinbock (1976), the distribution pattern of treponemal lesions on Hutten’s skeleton is characteristic of yaws. Additionally, as we will see, Hutten was in Greifswald on the Baltic coast when he experienced his first symptoms, coinciding with the recent paleogenetic discovery of yaws cases in the broader Baltic region, specifically two in Vilnius, Lithuania, and one in Turku, Finland.^25^ In this regard, and with all caveats, this study can contribute to the ongoing debate surrounding the nature and origin of this early modern treponemal epidemic.

## The anthropologist Erik Hug and the two Huttens

While the exact location of Hutten’s grave on Ufnau Island in Lake Zurich remained unknown for centuries, two possible individuals believed to be Hutten were discovered within a decade. Ufnau Island has been in the possession of Einsiedeln Abbey since 965. In 1958, during the renovation of the former parish church of St. Peter and Paul on the island, a grave containing the skeleton of an individual was unearthed, henceforth called Hutten-H58 (H58).^26^ During the demolition of a small transept added to the church in 1676, a skeleton was discovered positioned closely to the outer wall beneath a sandstone slab. Notably, near the church wall, an engraving reading ‘HVTTENVS’ came to light. This discovery led to the belief that the remains of Ulrich von Hutten had been found. To confirm this assumption, an assessment was commissioned by the Swiss anthropologist Erik Hug (1911–91). Although features consistent with Hutten were observed, no traces of syphilis were found in H58’s bones. Despite H58 being reburied under a monumental slab in the presence of descendants of the Hutten family, Hug expressed scepticism regarding whether this was the real Hutten.^27^ This justified doubt led Hug to conduct further exploratory excavations in 1968 near the church on Ufnau Island. Within a short period, he discovered the human remains of another individual, now referred to as Hutten-H68.^28^ In contrast to H58, this skeleton exhibited clear treponemal bone changes.

Hug embarked on the task of meticulously documenting his discoveries. Collaborating with the monastery’s in-house photographer, Father Damian Rutishauser (1931–2011), Hug produced a thorough photographic record of H68. Hug extended his efforts by assembling a team of medical specialists to conduct various examinations and cross-referencing Hutten’s descriptions with the skeletal findings. Following this comprehensive investigation, Hug concluded that H68 undoubtedly represented the remains of Ulrich von Hutten. Subsequently, Hug dedicated several years to delivering numerous public lectures in which he presented his evidence. Interestingly, and for reasons still unclear, Hug chose not to summarise his discovery in a formal scientific publication. The only printed materials were occasional, short, and rather superficial reports on his lectures.^29^ Hug passed away in 1991, bequeathing all his documents pertaining to Hutten to Einsiedeln Abbey. Nevertheless, these documents and photographs remained inaccessible, seemingly received and stored by Father Damian. It wasn’t until his demise in 2011 that these materials were rediscovered and documented by the Einsiedeln Abbey archives, beginning around 2012.^30^

## The ‘syphilitic’ and his records

From a medico-historical perspective, the case of Ulrich von Hutten presents a remarkable situation. He stands out not only as one of the earliest patients with treponemal disease who meticulously documented his main symptoms and the various attempts to cure his illness but also, with the discovery of his human remains in 1968, the way was paved for additional paleopathological comparisons. Hutten not only candidly discussed his illness in his extensive collection of surviving letters but also dropped occasional hints about it in his elegies and dialogues. Many references to the early stages of his infection can be found particularly in his *Querelae* against the Lötzes (1510). However, the most significant contribution to the history of medicine comes from his detailed account of the treatment he underwent with guaiacum wood in Augsburg during the autumn of 1518. This treatise was later published as *De guaiaci medicina et morbo gallico* [On the Guaiac Medicine and the French Disease] in Mainz in April 1519. Hutten not only chronicled his personal symptoms but also provided a detailed account of the treatment involving the novel guaiacum wood imported from tropical South America, which notably brought about temporary improvements in his health. Hutten’s treatise was soon translated into German (printed 1519, 1524, 1531), French (printed around 1520, 1530), and English (printed 1533, 1539, 1540). The demand for Hutten’s explanations persisted because, as Sticker (1909) demonstrated, several verbatim quotations and entire paragraphs were incorporated into many important sixteenth-century writings on syphilis without proper attribution to Hutten.^31^

Hutten has also captivated many modern authors. Oppenheimer (1902) translated the entire Latin text of *De guaiaci medicina* into German.^32^ While this translation is valuable, it tends to be highly interpretative in certain details and should therefore be approached with caution. Zimmermann (1932) and Benedek (1992) both delved into Hutten’s description of his symptoms.^33^ While Zimmermann’s translations display some idiosyncrasies, Benedek relies primarily on Oppenheimer. Both Benedek and Jillings (1995) specifically examine the metaphorical use of illness in Hutten’s writings.^34^ However, the most comprehensive study by far is Peschke’s (1985), which not only describes *De guaiaci medicina* and the associated guaiacum treatment but also places it within its historical context.^35^ To ensure an accurate interpretation, we have opted to translate Hutten’s texts directly from Latin.

## Willem F. R. Essed and yaws: a novel view on the French Disease

Hutten shared the prevailing belief that the French Disease emerged during the French army’s occupation of Naples in 1495 (HB 5, 399–400). Upon closer examination, the general symptoms of the French Disease, as described by Hutten, do not necessarily align with the modern characteristics of venereal syphilis but rather bear resemblance to yaws. By ‘modern symptoms’, we refer in this context to the clinical pictures as they have been consistently described since around 1900. As previously noted, this aligns with the hypothesis put forward by Dutch tropical physician Essed in his treatise *Over den Oorsprong der Syphilis* (1933), suggesting that yaws was the cause of the epidemic starting in the late fifteenth century.^36^ Born in 1893 in what was then the Dutch colony of Suriname in tropical South America, Essed possessed a unique background in tropical medicine and an early passion for microbiology, which would later shape his historical work.^37^ Essed had a wealth of firsthand experience, regularly observing and comparing the symptoms and partly severe manifestations of these diseases in his daily medical practice. This opportunity continued when he relocated to Java, by then part of the Dutch East Indies, in 1921, assuming the role of director in multiple laboratories and specialising further in parasitology. When Essed first became interested in the history of early syphilis, he quickly realised that the historical descriptions of the symptoms of the French Disease did not align with his observations and what he found in medical textbooks.^38^ This prompted him to delve deeper into the subject, meticulously examining the writings of late fifteenth- and early sixteenth-century authors. In this pursuit, he made extensive use of the textual tradition concerning the history of the French Disease, as detailed by Arrizabalaga et al. (1997).^39^ Notably, the Venetian physician Luigi Luigini (1526–after 1577) published an extensive two-volume collection of the earliest treatises on the new plague by fifty-nine authors in 1566/67.^40^ Two centuries later, in 1728, this collection received a reissue with annotations by the eminent Dutch physician and clinical teacher, Herman Boerhaave (1668–1738).^41^ Essed primarily relied on Boerhaave’s edition, complemented by smaller collections, such as that curated by the German professor of medicine, Philipp Gabriel Hensler (1733–1805), in 1783.^42^ In this manner, Essed analysed the earliest accounts of the epidemic from Italian, German, Spanish, and French sources, penned by chroniclers, physicians, and even patients.^43^ Thus, in his medical historical approach, Essed was able to rely on a broad textual foundation, with the majority being Italian sources. These sources were, for the most part, the same ones that Arrizabalaga et al., in their medical and social historical study on the French Pox, extensively referenced. Drawing from all his textual studies and comparative analyses, Essed ultimately concluded that the early French Disease was likely largely identical to yaws.

Before we delve into Ulrich von Hutten’s detailed descriptions, we will first provide a brief overview of the symptoms and the progression of yaws. According to *Manson’s Tropical Diseases*, 22nd edition (2009), early-stage yaws consists of primary and secondary stages.^44^ In the primary stage, typically occurring after an average incubation period of three weeks, a primary lesion (‘mother yaws’) appears at the entry site of the infecting organisms. These lesions commonly manifest on the legs, arms, face, and neck, presenting as round or oval papules with diameters ranging from two to 5cm. They may progress into large papillomas and are often itchy, leading to excoriation and ulceration. Primary lesions can persist for three to six months and can spread, leading to multiple lesions in other parts of the body.

Secondary lesions usually emerge within a few weeks to up to two years following the appearance of the primary lesion. They may be preceded or accompanied by symptoms like fever, joint pain, malaise, and generalised swelling of the lymph nodes. These skin manifestations resemble the initial lesion but are more widespread and may become crusted. Removing the yellow crust reveals granulomas resembling raspberries, earning yaws the alternate Latin name ‘*framboesia’* from the French ‘framboise’ for raspberry. Additionally, secondary lesions can take various forms, including annular, discoid, crescentic, or irregularly shaped papules and nodules. Hyperkeratotic plaques may develop on the palms and soles, making walking painful. Secondary lesions tend to appear in crops and can last for up to six months, sometimes healing without leaving scars. Bone involvement during the secondary stage is characterised by non-destructive osteitis and periostitis, with affected bones causing pain that worsens at night and tenderness.

After the secondary stage, there may be a complete remission or a latency of five or more years before progressing to the persistent tertiary stage of yaws. During this phase, individuals develop necrotic and destructive skin lesions, along with the formation of gummatous growths leading to scarring and deformity. Late-stage manifestations may also include the presence of fibrous nodules around the elbows and knees, disfiguring lesions in the nasopharyngeal region, and a characteristic bowing of the tibiae (‘sabre shin’).

Essed’s description of the symptoms of yaws broadly aligns with what has just been summarised.^45^ Due to his extensive literature studies in tropical medicine, Essed provides detailed insights into the course of yaws. Essed additionally mentions that during the incubation period, prodromal symptoms such as general lethargy and insomnia may occur. These symptoms are often accompanied by headaches and limb pain, which can be quite severe, with pain typically localised in the joints and long bones. The primary lesion can vary significantly in appearance but mostly presents as an ulcer of varying size. It is highly infectious and can persist for one to three months or even outlast the appearance of secondary papules. During the initial six months of the disease, it is common for patients to endure bone and joint pain, which tends to intensify during the evenings and at night. Throat pain and high fever can also manifest during this period.

The secondary stage, characterised by distinctive skin abnormalities, represents the principal clinical presentation of yaws and commences with a dry, scaly rash. This stage can endure for an extended period, ranging from a few months to many years, partly due to recurrences. The distinctive feature of fully developed yaws papules is their raspberry-like appearance, protruding above the skin and covered by a fairly thick yellow crust that can be painlessly removed. Once the crust is removed, the surface appears wart-like with a tough fibrous exudate that quickly coagulates. These papules also emit a distinct raw odour. Occasionally, the crust can take on a greenish, brown, or black hue due to underlying changes. Essed observed such papules most frequently at the corners of the mouth, in groin folds, the genital and anal regions, although they could appear anywhere on the body. These papules are usually painless, causing discomfort only when they ulcerate, sometimes forming deep incrustations that take months to a year to heal. The healing process results in the characteristic net-like scarring, with some areas darker and others lighter than normal skin. In some cases, a dry, raised, and rough scab covers these papules, particularly in facial areas, giving the affected individual a disfigured appearance. In stark contrast to syphilis, with yaws, inflammations of joints and bones can occur as early as in the second stage, causing significant pain, particularly in the evening and at night. These inflammations primarily affect the hands, knees, and feet.

Finally, Essed summarised the most prominent characteristics of tertiary yaws:^46^ 1. Regarding tertiary skin conditions, the ulcerated papules from the secondary stage can persist for years, potentially enlarging into large ulcers. Spontaneous development of superficial and deep ulcerative processes is common in the third stage. The deeper ulcers have a crater-like appearance with undermined edges. Both types of ulcers can spread over large areas, occur in multiple instances, and heal with significant scarring. These ulcers can affect any body part, including the face, chest, back, arms, and legs. 2. Notably, yaws is distinguished from syphilis by its frequent night pains, which can be intense. Joint disorders typically begin with severe joint pains, which often worsen at night to the extent that patients may have difficulty moving their limbs. 3. Joint and tendon sheath disorders may manifest with clear signs of inflammation, leading to joint destruction with stiffening and scar contractures. This can severely affect the elbow, knee, foot, finger, and hand joints. 4. Leg deformities are very common, often accompanied by night pains that precede inflammatory processes. Periosteal nodes are frequently observed in the radius, ulna, and tibia. Diffuse osteitis and periostitis particularly affect the tibia, causing significant thickening and sometimes forward convexity. 5. Another typical feature is the presence of hard and occasionally painful nodules found near joints, primarily at the elbows, near the heads of the femurs, heads of fibulas, and ankles. 6. In all regions affected by yaws, there are reports of mutilating inflammations in the nasopharyngeal region (rhinopharyngitis mutilans), resulting in severe and disfiguring facial destruction.^47^ 7. Yaws can also lead to the development of papillomas on the soles of the feet. These papillomas initially present as extremely painful swellings and eventually form thick callus layers on the soles, which can ulcerate and persist for many years.

Similar to yaws, venereal syphilis progresses through multiple stages. Transmission can also occur congenitally in children, which is not discussed here. According to *Rook’s Textbook of Dermatology*, 7th edition (2004), primary syphilis is characterised by the appearance of a hard, button-like ulcer known as a chancre, typically up to a centimetre in diameter.^48^ It may appear on any skin or mucous membrane surface where sexual contact has occurred, but it commonly manifests on the external genitalia. The sore is painless and may persist for up to three months if left untreated.

During secondary syphilis, generalised manifestations occur on the skin and mucous membranes, usually around eight weeks after exposure. Symptoms often include a coppery-red rash that does not itch and is symmetrically distributed. Papules rarely exceed 0.5cm in diameter. Additionally, hyperkeratotic lesions on the palms and soles may flake, peel, and fissure. Unlike in yaws, pustular ulcerative syphilides are not observed in present-day syphilis. Fever, headache, bone and joint pains, particularly pronounced at night, may also be present. Other systemic features may include neurological involvement, eye inflammation, and organ damage. Symptoms typically resolve within the first year of infection.

Tertiary syphilis may occur after a latency period of up to twenty years. Manifestations include tubercular syphilides, which are protruding, firm nodules on the skin, and gummata, granulomas that can appear anywhere on the skin, palate, or other organs. Gummata often exhibit central necrosis and ulceration with peripheral healing, resulting in tissue-paper scarring. Unlike yaws, gummata are usually painless even when they ulcerate. Late-stage syphilis can lead to severe cardiovascular and neurological complications.

This somewhat detailed outline of the present-day symptoms of yaws and venereal syphilis in all their stages will greatly help us to understand and reinterpret Hutten’s own perception of the epidemic and his own symptoms.

## Hutten’s general perception of the French Disease

In his works, Hutten provides valuable general information about the manifestations and course of the French Disease. When he wrote his treatise *De guaiaci medicina et morbo gallico* in the autumn of 1518, it appeared that the severity of the general disease had diminished over the two decades since its emergence. Thus, he was stating the following:

When it [the French Disease] first arose, its hideousness was so great that what now rages is scarcely considered of that kind: ulcers resembling the appearance and size of acorns, rough and protruding; a filthy fluid flowing from them; and such a great stench emitting that anyone who had come into contact with its odour would soon be believed to be infected. The pustules, ranging in colour from black to green, were more tormenting to the sufferers due to their appearance rather than causing pain, although they could be torturing when inflamed (HB 5, 402).^49^

For centuries, the French Disease had been equated with venereal syphilis. Given that the rash in the second stage of venereal syphilis often goes unnoticed and is non-itching in modern times, this passage has led to the question whether the virulence of the pathogen may have waned over time.^50^ Additionally, there have been speculations that the initial outbreak could have affected an immunologically unprepared population with no resistance, leading to a severe reaction.^51^ However, when we objectively analyse Hutten’s description, we can discern virtually the entire presentation of present-day yaws in its second stage. The protruding ulcers resembling acorns likely correspond to the aforementioned raspberry-like ulcers, which are closely related to yaws. Additionally, there is the viscous exudate with its characteristic odour, which evidently became unbearable due to poor hygiene. Even the overall painlessness and the colour of the pustules from greenish to black align with Essed’s description of yaws. Hutten then shifted his focus to the later and milder course of the disease, continuing:

What followed and now roams everywhere, appears more tolerable in its hideousness, for it consists of small ulcers, sometimes not much raised and hard, at other times, there is a widespread, winding, and creeping scab [serpens scabies] that covers the dry, scaly flesh (HB 5, 403).

This is a picture that is consistent with secondary yaws as well. This ‘widespread, winding, and creeping scab’ appears to correspond to the annular, discoid, crescentic, or irregularly shaped papules and papillomas described above, or to the dry, raised, and rough scab mentioned by Essed, which can cover the papules. Furthermore, as we have also seen above, atypical in syphilis, even the secondary stage of yaws can lead to sometimes very painful inflammations and swelling in the bones and joints. Hutten expresses it as follows, although elements of the tertiary stage may already be implied here:

The contagion is accompanied by these secondary afflictions, which exacerbate its severity. This ailment is so diverse that it seems to encompass a multitude of illnesses. Among its effects is a sharp pain in the joints, initially mild but progressively intensifying. Swollen limbs, some with collections and nodules that later become obscured, defy description in terms of the torment they conceal. This aspect of the disease is indeed the most dreadful, as it establishes itself as a stronghold within the body, persisting for extended periods and radiating various forms of agony throughout the entire body. The slower these swellings develop suppuration, the more intense the suffering. This torment mercilessly tortures and devastates its victims (HB 5, 405–6).

A sexually transmitted disease like venereal syphilis would typically involve infection exclusively through sexual practices. However, various accounts from early sources fundamentally challenge this mode of transmission. Hutten’s commentary greatly contributes to the clarification of this question.

At this time, it is believed that no one acquires this [disease], unless they have contaminated themselves through contact, which chiefly occurs during sexual intercourse. Hence, it less frequently affects children and the elderly or those inexperienced in sexual relations, more readily affecting those who are more lascivious and inclined toward sexual activity (HB 5, 403).

The crucial revelation in this quote is the recognition that the French Disease was transmitted through (skin) contact. Furthermore, in European regions where people typically wore ample clothing, transmission could particularly occur when couples engaged in direct skin-to-skin contact during intercourse. Another important point is that children, elderly individuals, and celibates could also acquire the disease, though less frequently, indicating that sexual activity was not a mandatory factor.

Hutten’s understanding of the mode of infection through contact aligns with the views of contemporary physicians, as demonstrated by Essed through his literary studies. In his *Tractatus de Morbo Gallico* [Treatise on the French Disease] (1498), the Ferrara professor of medicine, Sebastiano dall’Aquila (c. 1440–c. 1510), stated that infection could occur through various means, including coitus, sharing a bed, transmission from mother to child during suckling, or even through the air.^52^ Similarly, in his *De dolore in pudendagra* [On the pain in podagra] (1500), the papal physician Gaspar Torella (c. 1452–c. 1520) reported cases of infants being infected.^53^ Also, the Italian anatomist Niccolò Massa (1489–1569) in his *De Morbo Gallico* [On the French Disease] (1532), had observed children aged three, six, and eleven who had contracted the disease.^54^ Torella had further observed that any part of the body coming into contact with the virulent matter from the ulcers or pustules could become infected and start to rot.^55^ Additionally, Pedro Pintor (1423/24–1503), the personal physician of Pope Alexander VI and the entire Borgia family, noted in his book *De morbo foedo et occulto his temporibus affligente* [On the foul and hidden disease afflicting these times] (1500) that he had witnessed many cases of people contracting this disease through contact, especially during sexual intercourse.^56^ These sources and Hutten’s account collectively demonstrate that the method of transmission during the early years of the epidemic with the French Disease was by no means exclusively sexual.

## With lingering illness on the Baltic coast (1509–10)

As mentioned previously, in the autumn of 1509, the destitute Hutten embarked on a journey to the University of Greifswald, an ancient Hanseatic city located on the Baltic Sea. The initial support of the Lötze family culminated in a violent confrontation when Hutten attempted to proceed to Rostock in January 1510. Threatened with death and deeply humiliated, Hutten later sought to take revenge in his first major poem, the *Querelae* (1510), which consists of a series of lamenting elegies. Amidst his fervent polemics, Hutten also chronicled an initial bout of illness that plagued him for at least two years. Using his eloquent and poetic language, Hutten vividly portrays his dire condition characterised by severe fevers, accompanied by chills, aching limbs, profound fatigue, and a notable loss of appetite:

Behold, the violent quartan fever rages through my tender limbs, and all my strength has left my head; and where the trembling cold has exhausted my body, a heat no less fierce than Aetna’s fire surges within. Even the very force of the disease has struck the deepest marrow, and dry skin clings to the shaken bones. If I try to raise my feeble body on my feet, my legs falter under this slight burden; if I attempt to perform the usual tasks with my hands, my arms can hardly support the dry hands. Now my head rests on this shoulder, now it lies on that; there is no pleasure in drink, and no appetite for food (HB 3, 21).

A few pages later, Hutten reiterated that ‘in me, the quartan fever rages in alternating bouts’ (HB 3, 30). Due to this recurring fever, which manifested every fourth day, Benedek (1992) suggested that the clinical description was not consistent with syphilis but rather with malaria.^57^ Besides the possibility that Hutten’s ‘quartana’ may be a poetic trope for any irregular fever, Benedek overlooked the fact that Hutten also mentioned the presence of sores. It seems that primarily, there was a larger wound in Hutten’s flank or groin that filled him with fear, even causing him to dread imminent death.^58^ In addition to this primary lesion, there seemed to be other, presumably smaller wounds, hollow and dry ones, that Hutten perceived as excruciating.^59^ Apparently, these were pustules or ulcers covered with a crust. Moreover, the illness, with its lesions, presented itself as exceedingly painful, as Hutten described himself as ‘suffering from pain on all sides’ (HB 3, 32), and it deprived him of ‘both daylight and night-time rest’ (HB 3, 30).

What has just been described would align well with the second stage of yaws. The concerning sore in the groin could potentially be the primary infection that occurred in a typical location. This primary ‘mother yaws’ could have persisted until the appearance of the secondary papules, which could ulcerate (‘hollow wounds’) and become encrusted (‘dry wounds’). The severe general symptoms, including high fever and malaise, would also be consistent with yaws, and the possible bone involvement would be indicated by the aching limbs and the nocturnal pain. While the skin manifestations appear typical, it is possible that the fever indeed may have had a different origin. Hutten even provides a hint for an approximate timeframe for this severe period of illness, as noted in the *Querelae*:

I am not only oppressed by sickness and poverty. Winter has twice relented, summer has been completed as many times, and my health remains as it was before. Nor do I suffer any less; on the contrary, the disease grows with time, and the foul contagion has increased in strength through growth (HB 3, 22).

The *Querelae* were written in the spring 1510. According to Hutten, two winters and two summers had already elapsed. If we include the winter of 1509–10, the symptoms would have started in the spring of 1508. At that time, Hutten was enrolled at the University of Leipzig. It has often been assumed that it was this occasion when Hutten contracted syphilis.^60^ Assuming it was indeed syphilis, one would need to consider the incubation period (median twenty-one days), the duration of the primary infection (six to eight weeks), and the latency period (six to eight weeks) until the onset of the second stage. In this scenario, the infection would have occurred in the autumn or winter of 1507–8. However, with yaws, the primary papilloma or ulcer could have existed for some time without significant symptoms before its ulceration, the spread of secondary papillomas, and the onset of general symptoms.

In another passage within Hutten’s writings, there is a more specific reference that sheds light on the presumed secondary stage of his illness. In his dialogue titled *Febris prima*, penned in November 1518, Hutten engages in a direct conversation with fever personified.^61^ The fever reminds Hutten of their previous encounter eight years prior, visiting him every fourth day, but had only lasted for six months.^62^ In response, Hutten bitterly laments that he continued to suffer from constant illness even after the fever had subsided.^63^ These passages indicate that Hutten’s general symptoms with fever and extreme weakness endured for approximately six months. It is reasonable to assume that the secondary stage with multiple papillomas seamlessly transitioned into the tertiary stage, which involved bone manifestations, a progression that aligns with a possible course of yaws.

## The skeletal remains of a probable yaws patient

As previously mentioned, the human remains of two individuals were discovered on Ufnau Island, believed to be identical to Ulrich von Hutten. These individuals include the skeleton of Hutten-H58, which was found in 1958 near the former parish church St. Peter and Paul, and Hutten-H68, discovered by Erik Hug in 1968. As presented by Goujon et al. (2023), in 2016 an opportunity arose for a team from the Institute of Evolutionary Medicine at the University of Zurich, Switzerland, to re-exhume the skeletons of these two ‘Huttens’ and subject them to a series of comprehensive examinations.^64^ The primary objective was to conduct a comparative analysis between H58 and H68 to further substantiate the claim that the remains of H68 are indeed identical to those of Ulrich von Hutten. The average ^14^C radiocarbon age for H58 was determined to be 1403–43 cal AD [calibrated radiocarbon date for the Common Era], while H68 yielded a date of 1413–47 cal AD. Considering potential freshwater reservoir effects, both dates align with Hutten’s presumed lifespan. Macroscopic analysis suggested an age range of 40–60 years for H58, whereas for H68, the estimated age range of 20–30 years closely corresponds to Hutten’s age of 35 years at death. Microscopic examination of the dental cementum, possible only for H68, provided an even more precise estimate of 37 ± 3.5 years. The most surprising finding arose from the sex determination. While H58 exhibited clear male characteristics, anthropological examination of H68 revealed distinct female traits, further confirmed by the absence of Y chromosomes.

This observation correlates with H68’s elegant physique and stature ranging from 144–55cm. It raises the possibility of intersex misidentification, a phenomenon not uncommon in history.^65^ In such cases, individuals with intersex conditions, like congenital adrenal hyperplasia, were often falsely identified as male due to ambiguous genitalia and other secondary male sex characteristics.^66^ In fact, Hutten’s outward appearance seems to have aligned more with a delicate, possibly feminine constitution than with that of a powerful knight. He remarked that ‘nature has given me a gracile body’ (HB 5, 424). Similarly, the humanist Joachim Camerarius (1500–74), who knew Hutten personally, wrote that his body was ‘of a very small and weak form but possessed a huge and fierce spirit’ (HB 2, 362). Hutten’s parents also appear to have doubted his physical abilities, as they sent him to the monastery of Fulda at the age of eleven, ‘with the intention for me to stay there and become a monk’ (HB 2, 145). Typically, in a knightly family, the eldest son is chosen as the progenitor, destined to one day take over the castle and the lands.

Additional insights were obtained through archaeometric investigations. Analysis of stable isotopes of strontium and oxygen indicated a local origin for H58, whereas H68’s isotopic composition suggested a broader geographical origin in the Hesse region, consistent with Hutten’s known provenance. In contrast to H58 and other people from the upper class, stable isotopes of carbon, nitrogen, and sulphur in H68 indicated a less affluent diet with lower protein content. This finding is particularly significant in the case of Hutten, who, in the 19th chapter of his treatise *De guaiaci medicina et morbo gallico* under the heading ‘Praise for frugality against luxury’, vehemently criticised the drunkenness, gluttony, and excessive luxury of his German contemporaries, advocating instead for a simple lifestyle and diet (HB 5, 457–70). Despite Hutten’s multiple smear treatments with mercury, no significantly elevated mercury content was detected in the bones of H58 and H68. Finally, ancient DNA of *Treponema pallidum* was detected in the remains of H68. The search for subspecies was unfortunately inconclusive.

All these investigations, coupled with comparisons between the paleopathological findings and Hutten’s own medical report listed below, as well as the absence of other skeletons with treponemal alterations in the cemetery of St. Peter and Paul, provide compelling evidence for the identity of H68 as Hutten.^67^ Unfortunately, a genetic kinship analysis was unsuccessful, as Ulrich von Hutten’s male line diverged from the line of the still-living members of the Hutten family approximately twenty-five generations ago, with Hutten’s branch dying out in 1713.^68^

The skeleton of H68-Hutten is remarkably well-preserved, except for a few minor hand and foot bones. To enhance understanding and illustrate the noteworthy findings, we have created a dedicated website.^69^ Within this skeleton, we can distinguish findings attributed to treponematosis from those with different causes.^70^ Treponemal lesions were identified in the following locations: seventh cervical vertebra, right eleventh rib, right proximal ulna, distal left femur, right proximal tibia, left distal tibia, and both proximal and distal fibulae. Non-treponemal findings include an unclear hyperostosis and a frontal roughening of the skull, a likely traumatic angulation of the left humeral head resulting in humerus shortening, degenerative disc disease, the presence of Schmorl’s nodes in the tenth and eleventh thoracic vertebrae due to Scheuermann’s disease (juvenile kyphosis), and osteonecrosis of the right third metatarsal head (Köhler-Freiberg disease), likely acquired in late childhood.

Generally, there is a broad variety of bone manifestations in treponemal disease.^71^ They divide into two main categories: non-gummatous and gummatous lesions. Non-gummatous lesions involve periostitis, resulting in subperiosteal bone formation, plaque-like exostoses on the cortex, and a rough, hypervascular surface. Progressing deeper, osteoperiostitis and osteitis narrow the medullary cavity and obliterate it with sclerotic trabeculae. These non-gummatous manifestations often affect the tibia, fibula, clavicle, femur, ulna, and radius. Skull involvement can occur, particularly in the nasal, palatal, and frontal bones. Gummatous lesions, on the other hand, exhibit necrotising and proliferative reactions. Gumma formation involves a necrotic zone surrounded by collagenised connective tissue, resulting in thickened cortices, rough external surfaces, and surface depressions indicating foci of necrotic bone. These depressions are encircled by elevated areas reflecting reactive new bone formation. Gummatous lesions can also impact the nasal, palatal, and long bones throughout the body, moreover, it can lead to multiple skull destructions, known as ‘caries sicca’.

Many of the treponemal alterations mentioned above are evident in the skeleton of H68-Hutten. The skull appears relatively unremarkable, except for the unclear hyperostosis and frontal roughness, which might be an indication of beginning caries sicca. A probable spondylitis of the spinous process of the seventh cervical vertebra is rather inconspicuous. Additionally, a gummatous lesion can be identified on the right eleventh rib. The proximal right ulna exhibits an enormous gummatous swelling, while the distal left femur displays heavy plaque-like swelling and fistular furrows. The tibiae display signs of severe bilateral periostitis, leading to an irregular surface, osteitis, and distal medullary obliteration. Particularly, the distal diaphysis of the left tibia is thickened in a club-like manner and exhibits a complete occlusion with sclerotic trabeculae. Additionally, the middle part of the left tibia exhibits evident gummatous signs, characterised by lengthy necrotic surface depressions, accompanied by the formation of wall-like reactive new bone. The proximal right tibia features substantial plaque-like bone formation due to periostitis, while the middle area of the left tibia shows lengthy impressions. The back of the left tibia exhibits hyperostotic periostitis with furrows caused by blood vessels. Furthermore, the proximal fibulae display sunken necrotised areas, fistulae, and elevated strings of new subperiosteal bone formation, all typical characteristics of gummatous lesions. Lastly, the distal right fibula shows signs of another gumma.

All of these bone alterations are highly indicative of treponematosis, particularly the involvement of the tibiae. Thus, there is little doubt that Hutten-H68 suffered from such a disease. However, determining the specific type of treponematosis involved is a more nuanced task. According to Steinbock (1976) in his seminal textbook on paleopathology, the bone lesions observed in both yaws and syphilis exhibit remarkable similarities.^72^ Various types of lesions can be caused by both subspecies of *Treponema pallidum*, differing mainly in their quantitative frequency. To illustrate this concept, Steinbock created two skeletal drawings, shading affected regions differently.^73^ Solid black areas denoted the most frequent sites, while diagonal lines marked less common sites. In venereal syphilis, only the cranium and the tibiae are shaded black, with the cranium showing the highest incidence of osseous syphilitic lesions. Yaws, on the other hand, presents a different pattern. While the tibiae are also shaded black, there is an additional black shading of the distal femurs and the fibulae, which are also among the most frequently affected locations in yaws. In contrast to syphilis, the cranium is less affected. When comparing Steinbock’s schema for yaws with the locations of involvement observed in Hutten-H68, which include the intact cranium, the affected left distal femur, and both tibiae and fibulae, the correspondence is nearly perfect. This strongly suggests that Hutten-H68 was likely affected by yaws rather than syphilis.

## Hutten’s chronic symptoms versus Hutten-H68’s bone alterations

Hutten’s health never completely recovered after his time in the Baltic. During this period, he visited various spa resorts. For example, he stayed in Bad Ems in the spring of 1515 (HB 1, 30, 40). In fact, by October, he informed Erasmus that his trembling and his ailing foot had improved (HB 1, 102). Hutten also underwent a total of eleven treatments involving the topical application of metallic mercury, a standard approach for syphilis treatment for centuries to come (HB 5, 407–11). Although his condition occasionally showed signs of improvement, especially following an innovative therapy involving the consumption of guaiacum wood infusion during the Imperial Diet in Augsburg in autumn 1518, which renewed his hope.^74^ Even after experiencing that period of recovery, relapses were not far off. In any case, he had already visited spas again in the spring of 2019, specifically in Baden-Baden and Bad Wildbad (HB 1, 264, 273).

In his treatise *De guaiaci medicina et morbo gallico*, written in late 1518, Hutten documented the principal symptoms of his chronic illness. It is evident that Hutten suffered greatly, not only during the secondary stage but especially also in the tertiary stage with bone involvement. Night-time pains, a characteristic feature of yaws, were notably prominent. The following descriptions vividly portray the torments he endured:

Often, people shiver as if in a feverish state due to the severity of the pain. Abscesses also develop, which sometimes turn into cancer or fistulas, or they become long-lasting ulcers that frequently putrefy to such an extent that they first strip the bones bare, which then develop decay and are seriously damaged. Moreover, since the remnants of this disease are very persistent, people become so emaciated due to the constant suffering that the skin covers the innermost bones with all flesh consumed (HB 5, 406–7).

I was most severely afflicted by the disease, to the point where I could find no rest at night due to the pain and couldn’t even eat during the day (HB 5, 482).

Enumerating the affected body parts, Hutten began by describing a lesion on the inside above the left ankle, which eventually evolved into a persistent and severe suppurative inflammation of the middle part of the left tibia. This lesion had initially appeared eight years earlier, in 1510, likely marking the onset of the tertiary stage:

I had developed a peculiar bump [tuber] above the inner side of my left ankle, and once it had hardened into a callus, no ointment or poultice could soften it for a full eight years (HB 5, 406.5).

At first, my left foot had become useless due to the disease, which had already settled there for over eight years. And in the middle of the [left] shin, where the flesh is thinnest on the leg, there were ulcers accompanied by swollen, inflamed flesh, putrefaction, and immense pain. When one of them began to heal, another would immediately burst forth. There were indeed multiple ulcers scattered about, which the efforts of physicians could not reduce to a single one. Above those [ulcers], there was a bump [tuber] so hardened it appeared like bone, causing immense and excessive pain without interruption (HB 5, 483.15).

Indeed, this passage corresponds to a significant manifestation in the remains of H68. Specifically, it involves the pronounced thickening of the distal left tibia, coupled with the complete occlusion of the bone marrow space due to osteitis and evident signs of gumma formation in the middle part. As mentioned above, these multiple and sometimes confluent ulcers are typical of tertiary yaws. Hutten also reported a similar lesion on the right side, which occurred later and appeared to be less severe:

Just above the right ankle, there existed another accumulation, as hard as bone, representing the oldest remnant of the disease’s latest relapse. Despite the attempts of physicians to open it with both iron and fire, as well as all kinds of caustics, they made no progress. At times, it would swell intensely, accompanied by extreme pain, while at other times, it would subside and become more tolerable. It hurt less when the foot was moved close to the fire, yet it couldn’t endure being covered with multiple layers of clothing. The discharge from it was profuse, seemingly unquenchable, and whenever I put weight on my foot, it caused unbearable pain (HB 5, 484.1).

In the bones of H68, there is a distinct gummatous lesion evident in the distal right fibula, encompassing the entire tubular shaft. Essed considered Hutten’s description of being unable to stand on his right foot due to pain, attributing it to the presence of inflamed sheet-like papillomas on the soles of his feet, a characteristic feature of yaws (‘crab yaws’).^75^ However, this interpretation appears to be unlikely, the pain was rather caused by the inflicted ankle and the osseous inflammation. The subsequent statement made by Hutten, indicating that his right hip and knee had become stiff and unusable, could align with the severe alterations observed in the proximal right tibia and fibula of Hutten-H68:

Then, above [the right ankle], both hip and knee had become entirely frozen [i.e. stiff], with the thigh emaciated to extreme thinness and the flesh so diminished, that scarcely anything but skin seemed to cover the bone. Furthermore, both joints were so unsteady [luxata] that for a long time, I could only stand with great discomfort, and ultimately even no longer, when the corresponding buttock had completely atrophied (HB 5, 484.8).

As Essed noted, joint disorders are common in tertiary yaws, leading to difficulties in moving limbs. In the case of Hutten, intense regional inflammation in the knee area could potentially lead to complete impairment of the muscle and tendon apparatus, thereby affecting the functionality of the right knee. This, in turn, could have a reactive impact on the hip. Of course, a neurological dysfunction caused by chronic treponematosis could also have been involved. Furthermore, Hutten was notably distressed by a suppurative condition on his right chest, in the vicinity of his lower ribs:

And on the right side, just below the lowest rib, there was an ulcer, indeed without pain, but with a foul discharge of pus bubbling forth and a filthy flow of sanies, like an external narrow-mouthed fistula, with a large cavity inside; and above it, another lump again, as if there a bone had emerged from the rib (HB 5, 484.16).

Correspondingly, H68’s right eleventh rib exhibits a distinct gummatous lesion, precisely at the location of the suppuration mentioned by Hutten. The ‘narrow-mouthed fistula with a large cavity inside’ corresponds to the deep, crater-like ulcers with undermined edges described by Essed in tertiary yaws. Finally, we will examine a non-treponemal condition on the left shoulder that has received relatively little attention from commentators:

The left upper arm [humerus] was in pain, to the extent that the arm could no longer be lifted. The outermost part of the shoulder [scapula] had weakened and become numb, and in the middle of the [upper arm] muscle, there was a nodule the size of an egg, while the rest of the arm had become extremely thin down to the hand (HB 5, 484.13).

Among Hutten-H68’s two humeri, the left humerus stands out. While the right humerus measures approximately 28cm in length, the left is only about 26cm, with both bone shafts exhibiting nearly identical thickness. Furthermore, the left humeral head is tilted at an angle of approximately 45 degrees, an X-ray image revealing regular bone trabecula. The most likely cause of this condition is a fully healed proximal humerus fracture, which can result from direct trauma or falling on the elbow.^76^ The difference in length of approximately 2cm can be attributed to the loss of length due to the tilting of the head. Notably, Hutten’s description of a nodule in the muscle, the size of an egg, aligns with the clinical presentation of a biceps tendon rupture and the subsequent contraction of the biceps muscle to the middle of the upper arm. Such a rupture can be triggered by bone splinters stemming from the fracture. Hutten was obviously not aware of the trauma and thought it had to do with his illness.

In summary, we have established corresponding pathological findings in H68’s skeleton for five prominent symptom complexes described in Hutten’s medical history. Furthermore, as already mentioned, no other skeleton displaying treponemal manifestations was discovered during subsequent excavations on Ufnau Island. Consequently, we can convincingly assume that the remains of H68 are indeed those of Ulrich von Hutten.

## Hutten’s final days and fatal illness

After Hutten was labelled a heretic and faced the imposition of the imperial ban, he fled to Switzerland in November 1522. Initially, he journeyed to Basel, where Erasmus, his former idol, refused to welcome him. This ignited a literary dispute between the two markedly different individuals, representing one of the less illustrious episodes in German humanism.^77^ Ultimately, he reached Zurich via Mulhouse, where Zwingli provided refuge, as he did for many other religious exiles.^78^ On July 21, 1523, Hutten penned a letter to his long-time friend Eoban Hesse from Zurich, expressing his desire to withdraw from the tumultuous conditions of war and immerse himself in scholarly pursuits (HB 2, 253). Given Hutten’s fragile health, Zwingli suggested that he undergo a spa treatment in Pfäfers, renowned for its thermal springs and situated in the Alpine Rhine Valley near today’s Bad Ragaz. Zwingli himself had recuperated from a bout of plague there and had connections with the Prince Abbot of Pfäfers Monastery, Johann Jakob Russinger, a fervent advocate of the Reformation. But the treatment proved disappointing and brought no relief, as Hutten wrote to Zwingli at the end of July. The abbot had been very kind and intended to keep Hutten for an extended period. However, the summer was exceptionally cold and rainy. Additionally, the baths, with their small bathhouses, were situated in a narrow gorge and could only be reached at great peril. As Hutten’s closing statement suggests, Zwingli had likely already arranged further accommodation, though Hutten was not yet aware of its exact location:

I have spent some time in the baths, which were not very comfortable as they were not warm enough. It seems that this rest did little to improve my health, considering the effort and danger involved. However, I cannot fail to mention the kindness and generosity shown to me by the abbot… he earnestly asked me to stay with him for several weeks when I was leaving. He also provided horses and other supplies generously for the journey. He advised me to return to the baths on occasion, but now it seems that the continuous rain over the past few days, mixing with the bathwater, may have negated their benefits. Cold water has never been in short supply here, falling from the sky or flowing down from the rocks, and sometimes these torrents threatened to destroy our little dwellings… Also, inform me about the lodging you have arranged for me, for I would have proceeded there today had I not been uncertain about the direction (HB 2, 255).

As a former parish priest of Einsiedeln, Zwingli maintained excellent relations with the monastery there, which had owned Ufnau Island since 965. It is possible that Zwingli wanted to either hide Hutten on the island or protect himself from potential difficulties arising from Hutten’s restless spirit, especially considering that the Zurich Reformation was in a critical phase at the time. The main motive may have been the fact that the parish priest of Ufnau, Hans Klarer, nicknamed Schnegg (‘snail’), was known for his healing skills.^79^ Indeed, Heinrich Bullinger in the first volume of his *Reformation History* (finished 1573) reported that Hutten went to ‘Hansen Schneggen’ who was able to treat ‘Blattern vnd Laemy’ (pox and palsy).^80^ Accordingly, Hutten mentioned in a letter to Nicolaus Prugner on 1 August from Zurich that he would be staying ‘with a certain physician a few miles from here for several days’ (HB 2, 256). There were no indications of Hutten’s impending demise. In his letter to Hesse on July 21, he still exhibited his customary fervour in the pursuit of justice.^81^ Apparently, Hutten also continued working, as his handwritten marginal notes in his estate, preserved in the Zentralbibliothek Zürich, clearly indicate that he was revising some of his books.^82^ Likewise, in his letter to Prugner dated August 1, he exuded optimism about his imminent recovery, holding onto hope that God would soon resolve his difficulties, although mixed with a trace of fatalism.^83^ Even in his last letter to the Zurich City Council of August 15, no signs of frailty were apparent (HB 2, 257). Nonetheless, his life drew to a close a mere two weeks later.^84^

The most likely date of death, 29 August 1523, is most reliably reported in letters by Erasmus and Beatus Rhenanus who habitually were best informed.^85^ However, the lawyer Claudius Cantiuncula gives 30 August, and the St. Gallen chronicler Johannes Kessler, in his *Sabbata*, reports 25 August.^86^ There has been a persistent misinterpretation arising from a remark by Rhenanus in his letter. After discussing Pope Adrian VI’s severe throat ailment that led to his death, Rhenanus added: ‘Hutten died of a similar disease, as far as the difficulty of swallowing food is concerned’.^87^ Hutten apparently experienced difficulty swallowing before his death, but this should not be misconstrued as evidence of ‘severe’ swallowing problems, sore throat, or even ‘Schluckpneumonie’ (aspiration pneumonia) as the actual cause of his death, as has been repeatedly claimed.^88^ It was also widely assumed from early on that Hutten had succumbed directly to his ‘morbus Gallicus’. The Zurich theologian Johannes Stumpf, in his *Swiss Reformation Chronicle* (completed around 1535), noted Hutten’s death in ‘blatterbett’ (pox bed) and simultaneously stated that Hutten had been buried on Ufnau Island.^89^ Similarly, the St. Gallen physician and reformer Joachim Vadianus, who had encountered Hutten in Vienna in 1511, expressed certainty in his *Epitome* (completed in 1532) that Hutten had died of the French Disease.^90^ This conviction was also shared by the Zurich physician and polymath Conrad Gessner in his *Bibliotheca Universalis* (1545).^91^

Considering that Hutten had not lost his old enthusiasm in his final letters, and did not complain of any deterioration in his health, it seems unlikely that he died directly due to the consequences of his treponematosis. Instead, it is much more probable that Hutten succumbed to an intermittent acute illness. It is conceivable that he contracted a cold during the rainy summer, particularly under the unfavourable conditions at Pfäfers Spa. This cold might have developed into pneumonia, which, in advanced stages, can be accompanied by choking and swallowing difficulties, eventually leading to his death. Pneumonia was one of the most common causes of death among young individuals in the pre-antibiotic era.^92^

## Conclusion

Regarding the alarming epidemics that plagued Europe since around 1495, the humanist Ulrich von Hutten was among the early patients who detailed their symptoms of the new disease known as ‘morbus Gallicus’ or French Disease. His descriptions carry particular weight because they closely correspond to the findings from his presumed remains. Significant importance was placed on translating Hutten’s statements as precisely as possible from Latin to avoid premature interpretations. For a long time, it was widely assumed that this early modern epidemic was attributed to venereal syphilis. However, recent paleogenetic studies by Giffin et al. (2020) and Majander et al. (2020) suggest that genetically closely related yaws may have played a significant role alongside a longstanding coexistence with venereal syphilis. In fact, as early as 1933, the Dutch tropical physician Willem F. R. Essed realised that historical descriptions of French Disease closely resembled the modern symptoms of yaws rather than venereal syphilis. In the case of Hutten, based on his records and the paleopathological findings on his skeleton, this assumption appears to be confirmed. While retrospective diagnosis must be approached with caution, Hutten’s described symptoms indeed closely resemble the modern presentation of yaws or a very similar disease. It is also conceivable that Hutten was infected with the previously unknown *Treponema pallidum* lineage identified by Majander et al. (2020). In this sense, this study aims to contribute to the ongoing debate and still unresolved question regarding the origin and nature of this treponemal epidemic.

## Footnotes

1 For a history of the French Disease and its spread in Europe, see Jon Arrizabalaga , John Henderson , and Roger French , The Great Pox. The French Disease in Renaissance Europe (New Haven and London: Yale University Press, 1997); see also Claude Quétel, *History of Syphilis* (Oxford: Polity Press, 1990) (transl. of *Le mal de Naples: histoire de la syphilis* [Paris: Seghers, 1986]). Other names for the French Disease or Morbus Gallicus were ‘mal de Naples’, ‘great pox’, ‘böse Blattern’, ‘bubas’; it was named ‘syphilis’ by Girolamo Fracastoro in 1530.

2 For biographies, see David Friedrich Strauss , Ulrich von Hutten, 2 vols (Leipzig: Brockhaus, 1858), 2nd rev. edn (Leipzig: Brockhaus, 1871 [numerous reprints]); Hajo Holborn, *Ulrich von Hutten and the German Reformation*, transl. by Roland H. Bainton (Westport, CT: Greenwood Press, 1937 [repr. 1978]); Herbert Jaumann, ‘Ulrich von Hutten’, in Franz Josef Worstbrock (ed.), *Deutscher Humanismus 1480–1520, Verfasserlexikon*, Vol. 1 (Berlin and New York: de Gruyter, 2008), 1185–1237; Eckhard Bernstein , Ulrich von Hutten mit Selbstzeugnissen und Bilddokumenten (Reinbek bei Hamburg: Rowohlt, 1988).

3 On German humanism, see Lewis W. Spitz , The Religious Renaissance of the Humanists (Cambridge, MA: Harvard University Press, 1963 [repr. 2014]), on Hutten 110–29; Lewis W. Spitz , ‘The Course of German Humanism’, in Heiko A. Oberman and Thomas A. Brady, Jr. (eds), Itinerarium Italicum: The Profile of the Italian Renaissance in the Mirror of Its European Transformations (Leiden: Brill, 1975); Eckhard Bernstein , German Humanism (Boston: Twayne Publishers, 1983), on Hutten 116–28; James H. Overfield , Humanism and Scholasticism in Late Medieval Germany (Princeton, NJ: Princeton University Press, 2019).

4
*In Wedegum Loetz et filium eius Henningum querelarum libri duo* (Frankfurt/O 1510).

5

Marius Kraus , Ulrich von Hutten und seine Gegner. Humanistische Invektiven am Vorabend der Reformation (Baden-Baden: Ergon Verlag, 2022), 414–42.

6 On Hutten’s reception, see Wilhelm Kreutz , *Die Deutschen und Ulrich von Hutten*. Rezeption von Autor und Werk seit dem 16. *Jahrhundert* (Munich: Wilhelm Fink Verlag, 1984); Hans-Gert Roloff , ‘Ulrich von Huttens Wiederkunft zwischen Philologie und Ideologie des 19. und 20. Jahrhunderts’, in Helmut Koopmann and Frank Baron (eds), The Revival of the Renaissance in the Nineteenth and Twentieth Centuries (Münster: mentis Verlag, 2013), 137–59.

7 Eduard Böcking (ed.), *Ulrichi Hutteni… Opera quae reperiri potuerunt omnia*, 5 vols (Leipzig: Teubner, 1859–61 [repr. Aalen: Otto Zeller, 1963]); *Operum supplementum*, 2 vols (Leipzig: Teubner, 1869–70 [repr. Osnabrück: Otto Zeller, 1966]); hereafter, HB, Vols 1–7.

8 Roloff, *op. cit.* (note 6), 141–3.

9 Strauss, *op. cit.* (note 2).

10 Arrizabalaga, Henderson, and French, *op. cit.* (note 1), 1–19.

11

Laura J. McGough , Gender, Sexuality, and Syphilis in Early Modern Venice. The Disease that Came to Stay (New York: Palgrave Macmillan, 2010), esp. 9–16.

12 Quétel, *op. cit.* (note 1), 140–1.

13

Lola V. Stamm , ‘Yaws: 110 Years After Castellani’s Discovery of Treponema pallidum subspecies pertenue’, The American Journal of Tropical Medicine and Hygiene, 93 (2015), 4–6 25870417 10.4269/ajtmh.15-0147PMC4497901.

14 For an overview of the non-venereal treponematoses, see Lorenzo Giacani and Sheila A. Lukehart , ‘The Endemic Treponematoses’, Clinical Microbiology Reviews, 27 (2014), 89–115 24396138 10.1128/CMR.00070-13PMC3910905, https://doi.org/10.1128/CMR.00070-13. For a brief overview of all subspecies of *T. pallidum*, refer to Mary Lucas Powell and Della Collins Cook (eds), The Myth of Syphilis. The Natural History of Treponematosis in North America (Gainesville: University Press of Florida, 2006), 9–31 .

15 Powell and Collins, *op. cit.* (note 14), 37.

16

Willem Frederik Robert Essed , Over den Oorsprong der Syphilis. Een Kritisch-Historisch Epidemiologische Studie tevens ontwerp eener nieuwe Theorie (Amsterdam: H. J. Paris, 1933), xii + 328 + iv pp., https://resolver.kb.nl/resolve?urn=MMUBA08:000000896:pdf

17 To the best of our knowledge, only the following authors drew upon Essed’s work: Gundolf Keil and Willem Frans Daems, ‘Paracelsus und die “Franzosen”, Beobachtungen zur Venerologie Hohenheims. Teil I: Pathologie und nosologisches Konzept’, *Nova Acta Paracelsica*, 9 (1977), 99–151, esp. 117–20; Catrien G. Santing , ‘Medizin und Humanismus: Die Einsichten des Nürnbergischen Stadtarztes Theodericus Ulsenius über Morbus Gallicus’, Sudhoffs Archiv, 79 (1995), 138–49, esp. 139–408658589 .

18 Karen Giffin, Aditya Kumar Lankapalli, Susanna Sabin, et al., ‘A treponemal genome from an historic plague victim supports a recent emergence of yaws and its presence in 15th century Europe’, *Scientific Reports*, 10.9499 (2020), https://doi.org/10.1038/s41598-020-66012-x

19

Kerttu Majander et al., ‘Ancient Bacterial Genomes Reveal a High Diversity of Treponema pallidum Strains in Early Modern Europe’, Current Biology, 30 (2020), 1–16 32795443 10.1016/j.cub.2020.07.058, https://doi.org/10.1016/j.cub.2020.07.058

20 For an overview and additional references regarding the Columbian, pre-Columbian, and Unitarian hypotheses, see Powell and Collins, *op. cit.* (note 14), 33–7; Giacani and Lukehart, *op. cit.* (note 14), 95–6. For more comprehensive reviews of the state of the field, see Brenda J. Baker and George J. Armelagos, ‘The Origin and Antiquity of Syphilis. Paleopathological Diagnosis and Interpretation’, *Current Anthropology*, 29 (1988), 703–7; Powell and Collins (2005), *op. cit.* (note 14), 63–496; Kristin N. Harper et al., ‘The Origin and Antiquity of Syphilis Revisited: An Appraisal of Old World Pre-Columbian Evidence for Treponemal Infection’, *American Journal of Physical Anthropology*, 146, suppl. 53 (2011), 99–133, https://doi.org/10.1002/ajpa.21613; Brenda J. Baker , Gillian Crane-Kramer , Michael W. Dee , et al., ‘Advancing the Understanding of Treponemal Disease in the Past and Present’, American Journal of Physical Anthropology, 171, Issue S70 (2020), 5–41 10.1002/ajpa.2398831956996, https://doi.org/10.1002/ajpa.23988

21 See also Baker, Crane-Kramer, Dee, et al., *op. cit.* (note 20), 7–12.

22 Verena J. Schuenemann et al., ‘Historic *Treponema pallidum* Genomes from Colonial Mexico Retrieved from Archaeological Remains’, *PLOS Neglected Tropical Diseases*, 12, 6 (2018), https://doi.org/10.1371/journal.pntd.0006447; Giffin et al. (2020), *op. cit.* (note 18); Majander et al. (2020), *op. cit.* (note 19); Kerttu Majander et al., ‘Redefining the Treponemal History Through Pre-Columbian Genomes from Brazil’, *Nature*, 627, 8002 (2024), 182–8, https://doi.org/10.1038/s41586-023-06965-x

23

Jon Arrizabalaga , ‘Problematizing retrospective diagnosis in the history of disease’, Asclepio, 54 (2002), 51–70 17191369 10.3989/asclepio.2002.v54.i1.135.

24

Jean-Laurent Casanova , ‘Human Genetic Basis of Interindividual Variability in the Course of Infection’ Proceedings of the National Academy of Sciences of the United States of America, 112 (2015)10.1073/pnas.1521644112PMC469741526621739, E7118-27, https://doi.org/10.1073/pnas.1521644112

25 Giffin et al. (2020), *op. cit.* (note 18); Majander et al. (2020), *op. cit.* (note 19).

26

Linus Birchler , ‘Ulrich von Hutten und ein gallorömischer Tempel auf der Ufenau’, in Jahrbuch vom Zürichsee 1958/59 (Stäfa and Zurich: Th. Gut Verlag, 1959), 59–86.

27

Erik Hug , ‘Das fragliche Skelett des Ulrich von Hutten. Anthropologische Begutachtung des Grabfundes auf der Ufenau’, Bulletin der Schweizerischen Gesellschaft für Anthropologie und Ethnologie, 36 (1959–60), 34–46 , esp. 44.

28 On the discovery of the two skeletons, see Antoinette Goujon, ‘Examination of Two Late Medieval Skeletons from the Island of Ufenau SZ. An Interdisciplinary Approach to the Identification of Potentially Historically Relevant Human Remains’ (unpublished MA thesis: University of Zurich: 2022), 12–20.

29

Hermann Jung , ‘Die Lues des Ulrich von Hutten. Aufsehenerregende Untersuchungen eines Experten-Gremiums der Medizinischen Fakultät der Universität Zürich’, Der Hautarzt, 20 (1969), 334–64311653 ; Hermann Jung , ‘Die Lues am Hutten-Skelet. Die letzten Zweifel an der Identität des 1968 auf der Insel Ufenau im Zürichsee entdeckten Gebeine konnten von der Forschung behoben werden’, Der Hautarzt, 22 (1971), 509 4942400 ; Erik Hug , ‘Das Grab des Ulrich von Hutten’, Bulletin de la Société Fribourgeoise des Sciences Naturelles, 60 (1971), 100 ; [editor], ‘Das Grab des Ulrich von Hutten. Die Entdeckung seiner letzten Ruhestätte auf der Ufenau, von Erik Hug’, *Nova Acta Paracelsica*, 9 (1977), 96–8.

30 For Erik Hug’s estate regarding Ulrich von Hutten, see Klosterarchiv Einsiedeln, A.22/666–672, F3.22/3 (written estate); F3.22/2 (photographs); C.22/7–8 (X-rays); F1.22/29 (photographs of descendents of Hutten at reburial of H58 on 22 June 1959).

31

Georg Sticker , ‘Ulrich von Huttens Buch über die Franzosenseuche als heimlicher Canon für die Syphilistherapie im 16. Jahrhundert’, Archiv für Geschichte der Medizin, 3 (1909), 197–222 .

32

Heinrich Oppenheimer (transl.), Ulrich von Hutten’s Ueber die Heilkraft des Guaiacum und die Franzosenseuche (Berlin: Verlag August Hirschwald, 1902).

33

Ernest L. Zimmermann , ‘“The French Pox of that Great Clerke of Almayne, Ulrich Hutten, Knyght”’, Janus, 36 (1932), 265–82, 297–310; Thomas G. Benedek, ‘The Influence of Ulrich von Hutten’s Medical Descriptions and Metaphorical Use of Medicine’, *Bulletin of the History of Medicine*, 66 (1992), 355–75.

34

Lewis Jillings , ‘The Aggression of the Cured Syphilitic: Ulrich von Hutten’s Projection of His Disease as Metaphor’, The German Quarterly, 68 (1995), 1–18.

35 Michael Peschke, *Ulrich von Hutten (1488–1523) als Kranker und als medizinischer Schriftsteller*, Kölner medizinhistorische Beiträge, 33 (Cologne: F. Hansen, 1985).

36 Essed, *op. cit.* (note 16).

37 The available records about Essed’s biography are sparse, see C. Bonne, ‘In Memoriam Dr. W. F. R. Essed’, *Geneeskundig Tijdschrift voor Nederlandsch-Indië*, 80 (1940), 854.

38 Essed, *op. cit.* (note 16), 139–43. In addition to other textbooks, Essed also used the seventh edition of *Manson’s Tropical Diseases* (1921), which features a substantial chapter on yaws.

39 See Arrizabalaga et al., *op. cit.* (note 1), 4–8.

40 Aloysius Luisinus [Luigi Luigini], *De Morbo Gallico*, 2 vols (Venice 1566–7).

41 Aloysius Luisinus [Luigi Luigini], *Aphrodisiacus sive de lue venerea*, 2 vols in one, Herman Boerhaave (ed.) (Leiden 1728).

42 Philipp Gabriel Hensler, *Geschichte der Lustseuche, die zu Ende des XV. Jahrhunderts in Europa ausbrach*, Vol. 1 of one (Altona 1783).

43 Essed used a total of twenty-eight Italian, thirteen German, and three Spanish sources, see Essed, *op. cit.* (note 16), 158–226.

44

Gordon C. Cook and Alimuddin I. Zumla (eds), Manson’s Tropical Diseases, 22nd ed. (Elsevier Saunders, 2009), 1140–3.

45 Essed, *op. cit.* (note 16), 144–57.

46 Essed, *op. cit.* (note 16), 151–7.

47 After his doctoral thesis, Essed published a historical study on this condition: Willem Frederik Robert Essed , ‘De oudste beschrijving van den rhinopharyngitis mutilans, tevens een historisch bewijs, dat deze aandoening van framboetischen en niet van luetischen aard is’, Geneeskundig Tijdschrift voor Nederlandsch-Indië, 76 (1936), 563–80.

48
*Rook’s Textbook of Dermatology*, 7th edition, Tony Burns, Stephen Breathnach, Neil Cox, and Christopher Griffiths (eds), 4 vols (Blackwell Science, 2004), Vol. 2, 30.5–15.

49 All translations from Latin or other languages are conducted by the author unless otherwise specified.

50 Robert J. Knell, ‘Syphilis in Renaissance Europe: Rapid Evolution of an Introduced Sexually Transmitted Disease’, *Proceedings of the Royal Society B*, 271, Suppl. 4 (2004), S174–6 https://doi.org/10.1078/0018-442x-00037, S174.

51 For example, the introduction of measles and whooping cough to the Fiji Islands in 1874 and 1883, respectively, had catastrophic consequences, resulting in thousands of deaths, see Botho Scheube , The Diseases of Warm Countries. A Handbook for Medical Men, 2nd ed. (London: John Bale, Sons & Danielsson, 1903), 571–2.

52 Essed, *op. cit.* (note 16), 175; on dall’Aquila, see also Arrizabalaga et. al., *op. cit.* (note 1), 66–8.

53 Essed, *op. cit.* (note 16), 177; on Gaspar Tor[r]ella and Pedro Pintor, see Arrizabalaga et. al., *op. cit.* (note 1), 113–44.

54 Essed, *op. cit.* (note 16), 196; on Massa, see also Arrizabalaga et. al., *op. cit.* (note 1), 270–2.

55 Essed, *op. cit.* (note 16), 177.

56 Essed, *op. cit.* (note 16), 181–2.

57 Benedek, 1992, *op. cit.* (note 33), 357.

58 For the dreadful wound, cf. ‘a mortal wound… makes me face immediate dangers to life’ (HB 3, 22); ‘life and death both dwell within my limbs’ (HB 3, 42); for its location, cf. ‘a lively wound has attacked my groin [ilia]’ (HB 3, 30); ‘a lamentable wound clung to my side’ (HB 3, 32).

59 For the wounds, cf. ‘hollow wounds’ (HB 3, 22); ‘cruel wounds’ (HB 3, 23); ‘bearing those wounds’ (HB 3, 24); ‘dry wounds’ (HB 3, 42).

60 See, e.g. Jaumann, *op. cit.* (note 2), 1187.

61
*Febris. Dialogas Huttenicus* (Mainz, February 1519); see also Jaumann, *op. cit.* (note 2), 1212.

62 ‘Have you forgotten, how, eight years ago, I [the fever] made you learned, pious, and patient, when I was present every fourth day for a period not longer than six months?’ (HB 4, 33).

63 ‘But I [Hutten], during all those years after your departure, suffered from one illness after another without interruption’ (HB 4, 34).

64

Antoinette Goujon , Rouven Turck , Manuel Janosa , Urs Leo Gantenbein , and Martin Haeusler , ‘Hutten or not? A Re-examination of Two Late Medieval Skeletons from the Island of Ufenau SZ’, Bulletin der Schweizerischen Gesellschaft für Anthropologie, 25.2 (2019–23), 17–44 , https://doi.org/10.5167/uzh-238679; see also Goujon, 2022), *op. cit.* (note 28).

65 For a preliminary discussion, see Michael E. Habicht, Francesco M. Galassi, Joachim H. Schleifring, and Andreas G. Nerlich, ‘The Controversial Skeleton of Ulrich von Hutten: An Additional Note Pertaining to Syphilitic and Identification Matters’, *The American Journal of Medicine*, 136, 9 (2023), e190–2, https://doi.org/10.1016/j.amjmed.2023.02.024

66 See, e.g. Elisabeth Daae et al., ‘Sexual Orientation in Individuals With Congenital Adrenal Hyperplasia: A Systematic Review’, *Frontiers in Behavioral Neuroscience*, 14, 38 (2020), https://doi.org/10.3389/fnbeh.2020.00038

67 In 2007, extensive excavations were conducted around the church of St. Peter and Paul, revealing the skeletons, and in some cases only individual bones, of a total of 40 adults and 53 subadults. None of these remains showed signs of treponematoses, see Jakob Obrecht, ‘Freienbach - Insel Ufnau: Chronologie und Ergebnisse der archäologischen Ausgrabungen’, *Mitteilungen des historischen Vereins des Kantons Schwyz*, 111 (2019), 17–53, https://doi.org/10.5169/seals-864848, esp. 39–41.

68 Goujon et al., *op. cit.* (note 64), 39–40.

69 Urs Leo Gantenbein, *The Human Remains of Ulrich von Hutten*, https://www.paracelsus.uzh.ch/hutten.html

70 For an overview and short description of the paleopathological findings, see also Urs Leo Gantenbein, ‘A Famous Early Syphilis Patient: Paleopathological Findings in the Remains of the German Knight Ulrich von Hutten (1488–1523), Humanist, Poet, and Author of a Book on Syphilis’, poster presented at the *Evolutionary Medicine Conference: Interdisciplinary Perspectives on Human Health and Disease*, University of Zurich, Switzerland, July 30–August 1, 2015, https://www.paracelsus.uzh.ch/docs/Gantenbein_Hutten_2015.pdf

71 On treponemal bone manifestations, see Arthur C. Aufderheide and Conrado Rodriguez-Martín, *The Cambridge Encyclopedia of Human Paleopathology* (Cambridge, UK: Cambridge University Press, 1998), esp. 154–71; Donald J. Ortner , Identification of Pathological Conditions in Human Skeletal Remains, 2nd ed. (San Diego, CA: Academic Press, 2003), esp. 273–319.

72

R. Ted Steinbock , Paleopathological Diagnosis and Interpretation. Bone Diseases in Ancient Human Populations (Springfield, IL: Charles C Thomas Publisher, 1976), 143 .

73 Steinbock, *op. cit.* (note 72), 114, 144.

74 For the circumstances of syphilis therapy in Augsburg, see Claudia Stein, *Die Behandlung der Franzosenkrankheit in der Frühen Neuzeit am Beispiel Augsburgs*, Medizin, Gesellschaft und Geschichte, Beiheft 19 (Stuttgart: Franz Steiner Verlag, 2003).

75 Essed, *op. cit.* (note 16), 242.

76 I would like to express my gratitude to my colleague, Dr. Thomas Böni, an orthopedic surgeon and medical historian, for his valuable assessment of these findings.

77 On the controversy between Hutten and Erasmus, see Holborn, 1937, *op. cit.* (note 2), 188–200; Werner Kaegi , ‘Hutten und Erasmus. Ihre Freundschaft und ihr Streit’, Historische Vierteljahrschrift, 22 (1924–25), 200–78, 461–514.

78 On Hutten’s stay in Zurich, see Hans Gustav Keller, *Hutten und Zwingli* (Aarau: Sauerländer, 1952); Fritz Büsser, ‘Hutten in Zürich’, in *Ulrich von Hutten. Ritter, Humanist, Publizist, 1488–1523. Katalog zur Ausstellung des Landes Hessen anläßlich des 500. Geburtstages*, ed. by Peter Laub (Kassel: Hessischer Museumsverband, 1988), 337–43; Hans Ulrich Bächtold , ‘Ulrich von Hutten und Zürich. Eine Nachlese zum Hutten-Jahr 1988’, Zwingliana, 18 (1989), 12–9.

79 On Schnegg, see Diethelm Fretz , ‘Johannes Klarer, genannt Schnegg, der letzte Gastgeber Huttens’, Zwingliana, 4 (1927), 417–40.

80 On Hutten, see Johann Jakob Hottinger and Hans Heinrich Vögel (eds), Heinrich Bullingers Reformationsgeschichte, 3 vols (Frauenfeld: Ch. Beyel, 1838–40), Vol. 1, 113.

81 ‘Let there be a new and unheard-of protest against wickedness in broad daylight; let future generations see and know what kind of people opposed honesty, laws, justice, faith, and religion with crime and audacity’ (HB 2, 253).

82 Bächtold, *op. cit.* (note 78), 13–9.

83 ‘If fortune smiles upon me, you will share in every way; if not, you will share in the common fate… If I recover, there will be no need to blame Fortune, and I hope that God will eventually put an end to these difficulties as well’ (HB 2, 256).

84 On Hutten’s death, see also Hans Gustav Keller , ‘Huttens Tod’, Archiv des Historischen Vereins des Kantons Bern, 39 (1947–48), 185–206 .

85 Letter of Erasmus to Conrad Goclenius of 25 September 1523 (HB 2, 378); Letter of Beatus Rhenanus to Jakob Wimpfeling of 27 October 1523, in Joseph Knepper, ‘Kleine Funde zum elsässischen Humanismus’, *Zeitschrift für die Geschichte des Oberrheins*, 21 (1906), 40–9, esp. 48.

86 Letter of Claudius Cantiuncula to Agrippa von Nettesheim of 12 September 1523 (HB 2, 352); Ernst Götzinger (ed.), *Johannes Kesslers Sabbata. Chronik der Jahre 1523–1539*, 2 vols (St. Gallen: Scheitlin & Zollikofer, 1866–8), Vol. 1, 169.

87 Knepper, *op. cit.* (note 85), 48.

88 See Keller, 1947–8, *op. cit.* (note 84), 202. The ‘Schluckpneumonie’ as actual cause of Hutten’s death was first claimed by Heinrich Grimm, *Ulrich von Hutten. Wille und Schicksal* (Zurich and Frankfurt aM: Musterschmidt Göttingen, 1971), 134.

89

Ernst Gagliardi , Hans Müller , and Fritz Büsser (eds), Johannes Stumpfs Schweizer- und Reformationschronik, I. Teil, Quellen zur Schweizer Geschichte, 5 (Basel: Birkhäuser, 1952), 180.

90 Joachim Vadianus, *Deutsche historische Schriften*, ed. by Ernst Götzinger, 3 vols (St. Gallen: Zollikofer, 1875–79), Vol. 3, 184.

91

Conrad Gessner , Bibliotheca Universalis (Zurich: Christoph Froschauer, 1545), fol. 342.

92 See, e.g. Scott H. Podolsky , Pneumonia before antibiotics. Therapeutic evolution and evaluation in twentieth-century America (Baltimore, MD: The Johns Hopkins University Press, 2006).

